# Factors that influenced pregnant women with one previous caesarean section regarding their mode of delivery

**DOI:** 10.1016/j.amsu.2020.05.007

**Published:** 2020-05-18

**Authors:** Amer Sindiani, Hasan Rawashdeh, Nail Obeidat, Faheem Zayed, Ala”a A. Alhowary

**Affiliations:** a Department of Obstetrics and Gynecology, Faculty of Medicine, Jordan University of Science and Technology, Irbid, 22110, Jordan; b Department of Anesthesiology and Intensive Care, Faculty of Medicine, Jordan University of Science and Technology, Irbid, 21110, Jordan

**Keywords:** Caesarean section, TOLAC, ERCS, Vaginal delivery, VBAC

## Abstract

**Background:**

During the last decades, the rate of caesarean section is increasing and this can increase the mortality and morbidity. Up to one third of the caesarean sections are attributed to the elective repeat caesarean section (ERCS). This study aims to evaluate attitudes and factors affecting the choice of pregnant women with one previous caesarean section regarding their mode of delivery in their second pregnancy. By assessing these attitudes, this study can help the efforts in developing strategies to increase the rates of vaginal delivery.

**Material and methods:**

A cross-sectional design was conducted by a structured questionnaire on 166 pregnant women who had delivered once by caesarean section for their first pregnancy and were in the third trimester of their second pregnancy. Any women with an absolute indication for caesarean section was excluded. The study comprises women who attend the clinic at our center in Northern of Jordan. Proper statistical tests were performed to assess the association between the choice of delivery and selected demographic and clinical factors.

**Results:**

About 55.4% responded that they would choose ERCS (n = 92) and the remaining participants chose trial of labour after caesarean section (TOLAC) (n = 74). Fear of pain was the most common reason for choosing caesarean section, accounting for 55.4%. Interestingly, our study did not show a significant association between the mode of delivery and demographic factors, such as age, educational level and occupation. The single independent significant factor influencing patients’ choice that our study revealed was “being informed about the complications of TOLAC”. The choice of TOLAC was almost four times higher for those participants who had been informed about the complications, compared to those who had not been informed.

**Conclusion:**

Proper counselling is a main factor that affected the patients’ choice toward the mode of delivery. Proper pain management may encourage patients to choose TOLAC because fear of pain was a main reason that patients requested ERCS instead of TOLAC.

## Highlights

• Pregnants with a previous caesarean section has the right to choose TOLAC or ERCS.
• Pregnants should be aware about the risks and benefits for each mode of delivery.
• Proper counselling pain management are the main factors affect their choice.
• Knowledgeable pregnant women tend to choose TOLAC over ERCS.

## 1 Introduction

“There is no justification for any region to have a caesarean section rate higher than 10–15%”: this was the considered opinion of a panel of reproductive health experts at a meeting organized by the World Health Organization in 1985 [1]. However, despite the aforementioned recommendation, the rate of caesarean section worldwide has been increasing since then, in both the developed and developing countries [[2], [3], [4], [5]] and has even been over 40% in some places [[6], [7], [8]]. In Jordan, the situation is similar as the rate of caesarean sections has risen significantly over the last two decades, according to Al Rifai [9], who found that the rate of caesarean section had reached nearly 30% in 2012 while it was only 18% in 2002. Furthermore, Al Rifai was also concerned about some operations being performed without any clear medical indication, and this might be attributed to maternal requests for a caesarean section [10].

Opinions differ on why the rates are rising so rapidly despite the fact that caesarean deliveries are associated with higher rates of maternal mortality as well as maternal and perinatal morbidity [11,12], and that they are also more expensive than vaginal deliveries. Most researchers believe that the main causes of this rise are the continuous monitoring of the foetal heart during labour [13], the lack of experience in dealing with instrumental delivery [14], the lack of experience in vaginal breech delivery, and maternal request [[15], [16], [17]]. Repeat caesarean section after a previous one is also a major contributing factor, accounting for more than one-third of all caesarean deliveries in the United States [18].

Despite recommendations from American College of Obstetrics and Gynaecology (ACOG) and Royal College of Obstetrics and Gynaecology (RCOG) to encourage all women who have previously delivered by lower segment caesarean section to attempt the trial of labour (TOLAC) as a safe option [19,20], there is nowadays a general tendency to adopt elective repeat caesarean section (ERCS) by both obstetricians and patients [7,[21], [22], [23], [24]]. For example, the overall vaginal-birth-after-caesarean-section (VBAC) rate in the United States declined sharply from 24% in 1996 to 8% in 2010; which is very low [25]. This reduction of the number TOLAC was explained in one study by the lack of audit system and the lack of practice guidelines [26]. Another factor is the variation in the success rate reported by different studies, the success rate ranged from as low as 27.4%–53.6% [27,28] to as high as 79.6–83.5% in many studies after careful selection [29,30]. Other factors for the low rate in low-income countries include delay in access to health care service, unavailability of painless labour, lack of constant availability of operating rooms in cases of emergency, poor educational status, great number of cases with unknown previous uterine scar, and poor record keeping of previous caesarean delivery [26]. Moreover, obstetricians might also discourage women who have had a previous caesarean section from choosing vaginal birth to avoid risks [31].

There are many factors that might affect their attitudes toward the mode of delivery, such as the fear of the consequences of vaginal birth, their level of education, their socio-economic status and their health care providers' opinions [32]. In Jordan, this is an updated study to assess maternal attitudes toward the mode of delivery in women with a previous caesarean section. Also, this study aims to identify the factors that may have influenced their choices. We sincerely believe that this study will help to devise better strategies to reduce caesarean section rates.

## 2 Material and Methods

### 2.1 Setting

This study was conducted at a tertiary care center. After obtaining approval from the Institutional Review Board approval, we invited and interviewed women who underwent one previous caesarean section to assess the factors and attitudes that would influence their decision regarding the mode of the delivery in their second pregnancy. The study was conducted between June and December 2018 in an observational analytical cross-sectional design. The confidentiality of the participants was protected by providing a code number for each participant during the data collection and analysis phases of the study. All women were interviewed in the outpatient clinics. Also, all women who had delivered only once (parity 1) by a caesarean section and were in the third trimester of their second pregnancy were included in the study. In addition, any women with an absolute indication for caesarean section was excluded from the study (examples include placenta previa, previous classical caesarean, high order multiple pregnancy and pelvic abnormality). All women underwent their previous caesarean section in our unit with good care and monitoring.

Our center is located in Northern of Jordan and provides the care for a large population from different social backgrounds, and for both urban and rural populations. In addition, the center provides the care for the population of all financial levels because most of the patients have the health insurance.

### 2.2 Data collection

The structured interviews were done by two senior residents and the data was collected using a structured questionnaire. The structured questionnaire includes demographic variables (age and monthly income), as well as clinical variables such as indications of the first caesarean section, whether the operation was an elective or emergency one, the type of anaesthesia used, and any complications during or after the caesarean section. In addition, the choice of delivery whether TOLAC or ERCS, the reasons for their choices, and the source of their information (doctors, media, friends) were investigated. Moreover, they were assessed if they had been informed about the complications of a trial of labour after a caesarean section and about the complications of repeated caesarean sections. The utilized tool in this study was derived from different literature sources. Face validity was conducted by four experts in the field and the results clearly indicated that the measuring tool used in our study was valid. Content validity was also assessed by three other experts and the Content Validity Index was 85%. The questionnaire was also clear and understandable for all participants.

### 2.3 Statistical analysis and sampling

Statistical analyses were performed using IBM SPSS Statistics Software (v. 21). Data were presented as frequency distributions for categorical variables and mean ± standard error of the mean for continuous variables and recorded in a spreadsheet; the significance level was 0.05. The Pearson χ^2^ test was used to investigate the differences in categorical variables between groups. Student's *t*-test was performed to examine the significance level of continuous normally distributed variables. Binary logistic regression was applied to study the contributory effect of different independent variables on the choice.

Before that, a pilot study was conducted using fifteen patients who possessed the same inclusion criteria, and these fifteen were not included in the final sample size. The results showed there had been no problems during collection, coding and analysis.

The sampling method includes the random selection of women who attend the obstetric clinics at our center which provides the care for a large population from different social backgrounds. The size was calculated according to the power of analysis formula with the following assumptions: power of 0.9, alpha of 0.05, beta of 0.1, the anticipated incidence for the ERCS group of 65% and the anticipated incidence for group who will choose TOLAC is 35%. According to that, the total required sample size is 112.

## 3 Results

### 3.1 Sample characteristics

One-hundred and sixty-six women participated in this study. The mean age of the women was 28.2 years with a range of 19-to-41 years old. Around 52% of the participants were unemployed (n = 86), 84% held a university certificate (n = 140) while the remaining 16% had graduated from a secondary school or less. (n = 26).

Furthermore, the results showed that almost 85% of the sample (n = 140) have no history of fertility treatment. The previous mode of delivery for all participants was caesarean section. Also, more than two thirds of the women had delivered through emergency caesarean section (n = 114), while the remaining by elective caesarean section. The general anaesthesia was conducted in more than two thirds of the women (n = 107). The mean birth weight for the new-born babies in the first delivery was 3.05 kgs. and ranged from 0.9 to 5 kgs.

Moreover, 83% of the participants did not experience any antenatal complications during their second (current) pregnancy. Also, only 13% of the participants were admitted to the hospital during their second (current) pregnancy (n = 22).

In addition, the findings showed that the treating physicians were the main source of information about the labour and delivery and accounted for 48% (n = 81), while 33% of the participants received their information from family and friends (n = 55) and only 18% received the information from the media. In addition, the results showed that almost half of the participants were informed about the complications of ERCS (n = 82). On the other hand, only 30% of the participants were informed about the complications of TOLAC (n = 49). The majority of the sample had plans for a future pregnancy (n = 143). Sample characteristics, namely, demographics and the clinical factors for participants, are presented in Table 1.

**Table 1:** Sample Characteristics: demographics and the clinical factors for participants (N = 166).

Categorical variables	N	%
**Numerical variables**	Mean ± SD	
**Age** (Years)	28.20 ± 4.36	
**Birth weight of first new-born** (in kilograms)	3.04 ± 0.61	
**Employment**		
Employed	80	51.80
Unemployed	86	48.20
**Educational level**		
Secondary education or less	26	15.70
Bachelor's degree and above	140	84.30
**History of fertility treatment**		
Yes	26	15.70
No	140	84.30
**Urgency of previous CS**		
Elective	52	31.30
Emergency	114	68.70
**Type of anaesthesia used in previous delivery**		
General	107	64.50
Regional	59	35.50
**Foetal outcome in previous delivery**		
Male	84	50.60
Female	69	41.60
Multiple	13	7.80
**Antenatal complications during the second pregnancy**		
Yes	29	17.50
No	137	82.50
**Previous admissions during the current pregnancy**		
Yes	22	13.30
No	144	86.70
**The main source of information about delivery**		
Treating physicians	81	48.80
Family and friends	55	33.10
Media	30	18.10
**Being informed about the complications of ERCS**		
Yes	82	49.40
No	84	50.60
**Being informed about the complications of TOLAC**		
Yes	49	29.50
No	117	70.50
**Planning for future pregnancy**		
Yes	143	86.10
No	23	13.90

Abbreviations: SD: Standard Deviation, N: Number, ERCS: elective repeat caesarean section, TOLAC: trial of labour after caesarean section.

### 3.2 Attitude of participants towards the choice of delivery

**Fig. 1:**
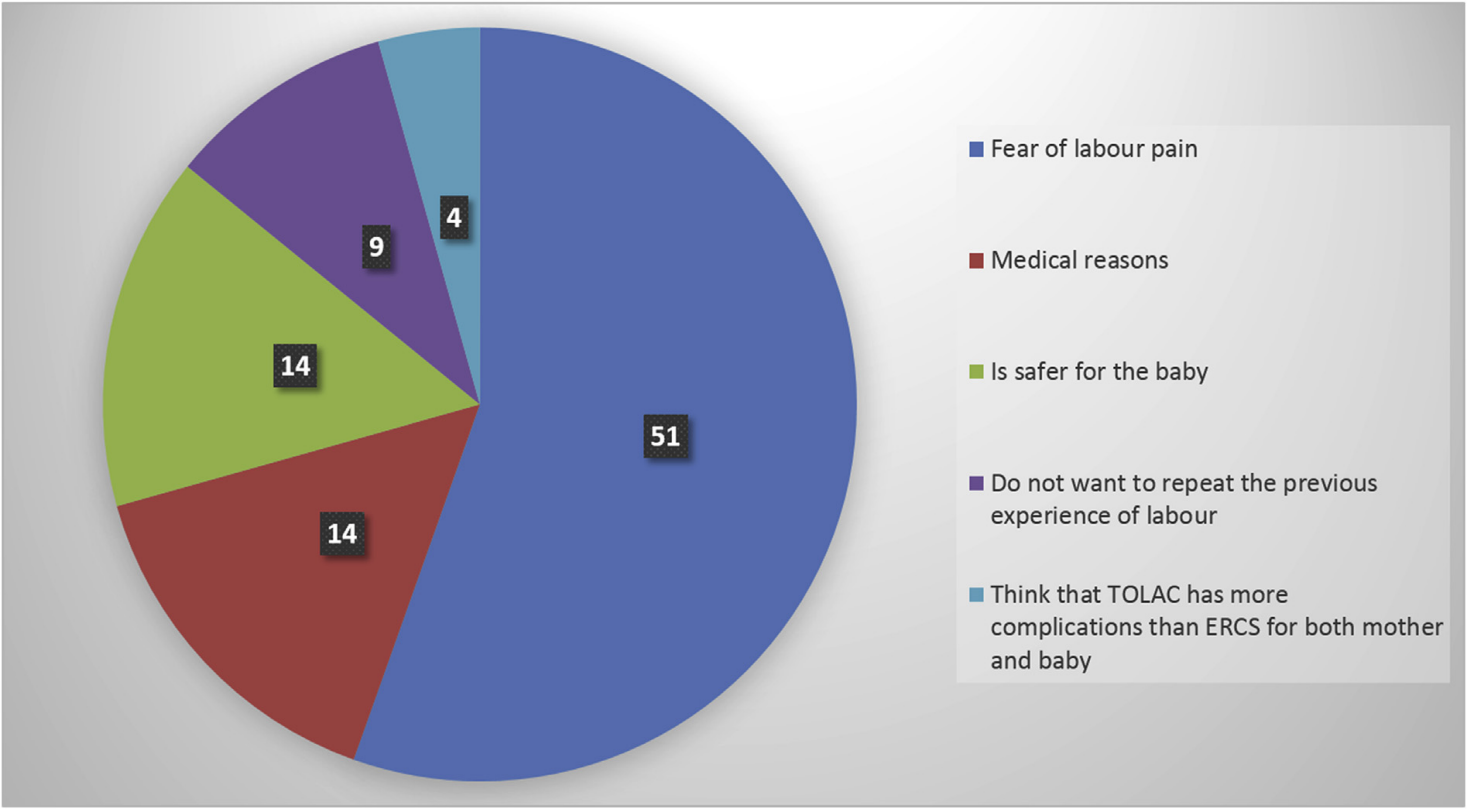
Attitudes towards choosing ERCS (N = 92).

Participants were asked about their choice of delivery for their second pregnancy. More than half of them (55.4%) responded that they will choose ERCS (n = 92) and the remaining participants said they will opt for TOLAC (n = 74). The attitude of participants towards the choice of delivery was assessed according to the different reasons given by them. Regarding those participants who decided ERCS, the main reason was the fear from labour pain, accounting for 55.4% (N = 51 out 92). Also, 15.2% (n = 14 out 92) indicated that there was a specific medical reason for their choice (these medical disorders are not indications for caesarean section; as hypertensive disorders, diabetes, mild renal impairment, and multiple sclerosis which require the induction of labour). Another 15.2% (n = 14 out 92) claimed that ERCS was safer than TOLAC. About 9.8% (n = 9 out of 92) did not want to repeat their previous experience (of failed-to-progress in labour) and 4.3% believed that TOLAC involved more complications than ERCS Fig. 1.

**Fig. 2:**
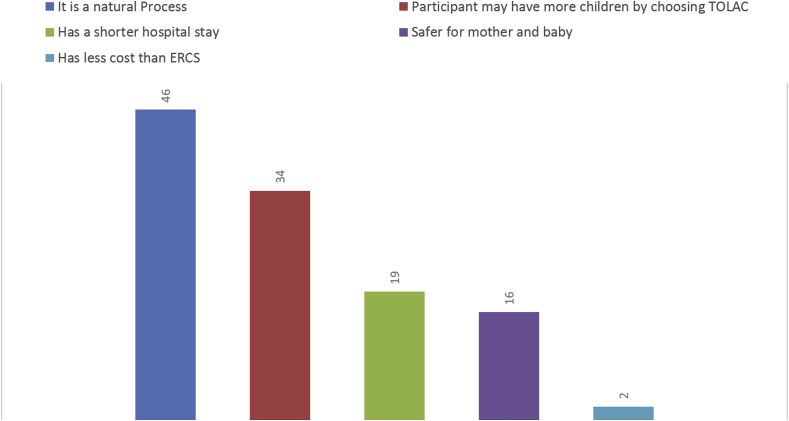
Attitudes towards choosing TOLAC (N = 74).

The attitude of participants towards their choosing TOLAC was also assessed using the various reasons they had given for their choice. Some participants had chosen more than one option at the same time. The most important reason given by the participants for choosing TOLAC was its natural process; this accounted for 62.1% (n = 46 out of 74). The second most important reason was that the participants believed that they might be able to have more children in the future if they achieved vaginal delivery this time; this accounted 45.9% of the participants (n = 34 out of 74). Also, 25.7% of the participants believed that TOLAC resulted in a shorter hospital stay (n = 19 out of 74), and 21.6% of the sample claimed that TOLAC was safer than ERCS (n = 16 out of 74). The economic reason was the least important reason and only 2.7% of the sample said that TOLAC costing less than ERCS (n = 2 out of 74). The choices for delivery and the attitudes toward the choice of delivery are presented in Figs. 1 and 2.

### 3.3 Factors affecting the choices of delivery

There was no significant association between the choice of delivery and the following variables: age, employment status, educational level, history of fertility treatment, level of urgency of the previous caesarean section (elective versus emergency), antenatal complications during the second (current) pregnancy, the source of information, being informed about possible complications with ERCS and birthweight of the previous pregnancy.

On the other hand, the results revealed that there was a significant association between the choice of delivery and "the previous admission during the second (current pregnancy)". Women with previous admissions were more likely to choose ERCS (18.5%, n = 17 out of 92) than to choose TOLAC (6.8%, n = 5 out of 74) (*P = 0.03*). The causes of these admissions did not include any absolute maternal or fetal indication for CS.

In addition, the results revealed that there was a significant association between the choice of delivery and "being informed about the complications of TOLAC", women who were informed about the complications of TOLAC were more likely to choose TOLAC (43.2%, n = 32 out of 74) than to choose ERCS (18.5%, n = 17 out of 92) (*P = 0.001*). The informed complication was a 0.5–1% rate of uterine rupture.

Moreover, a significant association between the choice of delivery and planning for a future pregnancy was found. Women planning for future pregnancy were more likely to choose TOLAC (93.2%, n = 69 out of 74) than to choose ERCS (80.4%, n = 74 out of 92) (*P = 0.02*). Table 2 summarises different factors that would influence the choice of the delivery.

**Table 2:** Factors affecting the choice of delivery.

Demographic and clinical variables	Choice of delivery		*P value*
	TOLAC n = 74	ERCS n = 92	
**Employment**			NS
Employed	34 (45.90%)	46 (50.00%)	
Unemployed	40 (54.10%)	46 (50.00%)	
**Educational level**			NS
Secondary education or less	10 (13.50%)	16 (17.40%)	
Bachelor's degree and above	64 (86.50%)	76 (82.60%)	
**History of fertility treatment**			NS
Yes	10 (13.50%)	16 (17.40%)	
No	64 (86.50%)	76 (82.60%)	
**Urgency of previous CS**			NS
Elective	20 (27.00%)	32 (34.80%)	
Emergency	54 (73.00%)	60 (65.20%)	
**Antenatal complications during the current pregnancy**			NS
Yes	11 (14.90%)	18 (19.60%)	
No	63 (85.10%)	74 (80.40%)	
**Being informed about the complications of ERCS**			NS
Yes	36 (48.60%)	46 (50.00%)	
No	38 (51.40%)	46 (50.00%)	
**The main source of information**			NS
Treating physicians	32 (43.20%)	49 (53.30%)	
Family and friends	28 (37.80%)	27 (29.30%)	
Media	14 (18.90%)	16 (17.40%)	
**Admissions during second pregnancy**			0.03
No	69 (93.20%)	75 (81.50%)	
Yes	05 (06.80%)	17 (18.50%)	
**Being informed about the complications of TOLAC**			0.001
Yes	32 (43.20%)	17 (18.50%)	
No	42 (56.80%)	75 (81.50%)	
**Planning for future pregnancy**			0.02
Yes	69 (93.20%)	74 (80.40%)	
No	05 (06.80%)	18 (19.60%)	
**Age** (Mean ± SD)	28.0 ± 3.8	28.4 ± 4.8	NS
**Birth Weight of the first pregnancy** (Mean ± SD)	3.0 ± 0.63	3.1 ± 0.59	NS

Abbreviations: SD: Standard Deviation, N: Number, ERCS: elective repeat caesarean section, TOLAC: trial of labour after caesarean section, NS: not significant.

To examine the predictors that may contribute to the choice of delivery, a binary logistic regression was applied (Table 3). The following predictors were entered into the equation of regression: age, occupation, educational level, history of fertility treatment, level of urgency in previous caesarean section (elective or emergent), complications during the current pregnancy, antenatal admissions during the current pregnancy, complications after first caesarean, being informed about the complications of ERCS, being informed about the complications of TOLAC, the source of information, and planning for future pregnancy. The results indicated that the full model had significantly predicted the odds for the choice of delivery (*P < 0.001*). After controlling for all factors, "informing patients about the complications of TOLAC" was the single independent variable that would affect the choice of delivery (*P = 0.001*). The odds ratio for informing patients about the complications of TOLAC was 3.7, indicating that the choice of TOLAC was almost four times higher for participants who had been informed about the complications compared to those who had not been informed.

**Table 3:** Logistic regression analysis to identify the significant predictors of the choice of delivery for women in the study (N = 166).

Predictors	B	Wald	P	Odds ratio	Lower CI-Upper CI
Age	−0.007	0.03	0.87	0.99	0.91–1.08
Employment	−0.092	0.07	0.80	0.91	0.45–1.85
Educational level	0.225	0.19	0.66	1.25	0.46–3.41
Treatment of infertility	0.215	0.20	0.65	1.24	0.48–3.20
Urgency of CS (either elective or emergent)	0.157	0.01	0.68	1.17	0.55–2.50
Complications after first caesarean	−0.021	0.60	0.97	0.98	030–3.20
Antenatal complications during current pregnancy	−0.055	0.01	0.91	0.95	0.33–2.68
Patient's source of information	−0.338	0.40	0.52	0.71	0.25–2.01
Admissions during this pregnancy	−0.770	1.47	0.22	0.46	0.13–1.60
Planning for future pregnancy	-.960	2.43	0.11	0.38	0.11–1.28
Being informed about the complications of ERCS	−0.270	0.35	0.44	0.76	0.38–1.52
Being informed about the complications of TOLAC	1.30	10.81	0.001*	3.7	1.70–8.05

Abbreviations: CI: confidence interval, ERCS: elective repeat caesarean section, TOLAC: trial of labour after caesarean section.

## 4 Discussion

Pregnant women with one previous caesarean section should be properly counselled about the mode of delivery during their antenatal visits, and this counselling has to be documented clearly in their medical records. They should be informed that the rate of success for vaginal birth after a caesarean section is about 70%, according to RCOG and ACOG.

Shared decision-making regarding the mode of delivery following a previous caesarean section is a relatively new concept in Jordan; it is still unclear whether our patients chose the mode of delivery based on a clear understanding of the benefits and risks of TOLAC and ERCS. Our study found that nearly half of our participants received their information about labour and delivery from the physicians treating them. In other words, about half of the participants gained their information from unprofessional sources, which probably provided them with misleading facts. This means that our current practice is deficient and is against the recommendations, which clearly state that every single patient should be counselled thoroughly by her physician about the benefits and risks of TOLAC and ERCS. Our study also showed that not all the participants who received their information from their physicians were sufficiently aware of the risks and benefits to be in a position to actively share in the decision-making process, as only 30% of the participants had been informed about the complications of TOLAC. We believe that this is a serious situation and can even lead to dangerous outcomes, if left unnoticed and not corrected. Therefore, adopting the idea of introducing a clear written consent form for patients with a previous caesarean section could be an effective way of ensuring that every patient has received the required information, clearly and comprehensibly delivered, before making a safe decision. This is reflected by the homogeneity of our sample, for example 85% held a university certificate, and 85% didn't receive fertility treatment which in turn reflects the level of education in the community regardless the social and finical status. This homogeneity reflects that these factors; as the level of education; did not influence or explain the increasing rate of ERCS over TOLAC. On the other hand, this emphasises the importance and necessity of patients' education and counselling about TOLAC and ERCS to raise the rate of TOLAC regardless if the pregnant woman has a university certificate or not.

Our study showed that 55% of the participants showed a positive attitude towards ERCS, which was comparable to the results from a similar study conducted in Kenya on patients with a previous caesarean section, which was 67% [33]. More than half of our participants opted for ERCS because of their fear of labour pain, which is consistent with other studies carried out in South Korea (2004) and Ghana (2008), which evaluated women's attitudes towards the mode of delivery and found that the fear of pain scored the highest positive response towards a caesarean section [34,35]. Therefore, we believe that increasing the maternal knowledge about pain-relief methods during labour, such as epidural analgesia and providing well-trained medical staff to optimize the obstetrical anaesthesia, may result in reduced maternal fear of pain and thus encourage more mothers to prefer vaginal delivery, as has been claimed by the American Society of Anaesthesiologists [36].

On the other hand, about 45% of the participants showed a positive attitude towards TOLAC, which is close to what was found in the Kenyan study as nearly a third of them opted for TOLAC [33]. The most important reason that influenced our participants to opt for vaginal birth was because it was a natural process; this accounted for about 62%. The perception of vaginal delivery as a natural process was also found to be the main positive response towards vaginal birth in other studies carried out in Kerman, Iran (2005) and in India (2017). In these studies, however, the women were questioned during their regular antenatal care sessions, and the specific focus was seemingly not on women who had previously delivered only by caesarean section as it was in our study [37,38]. Our study did not show a significant association between the mode of delivery and demographic factors, such as age, educational level and occupation; this finding was also consistent with the Kenyan study [33].

It is also important to highlight that there is another crucial factor that can affect the success rate of TOLAC which is the lack of clinical guidelines and the relative variance in TOLAC indication criteria. This is obvious by the high success rate of one study that reached 91% after considering the appropriate criteria [39].

The main strengthen point is that this study is the first conducted study to assess maternal attitudes toward the mode of delivery. In addition, the utilization of systematic conductance of well-prepared questionnaire is another strengthen point. Also, it sheds the light on important issue regarding the proper counselling of the patients. On the other hand, the relatively small sample size is a limitation. Moreover, conditions that may occur just before the delivery and can change the attitude of the women were not assessed.

## 5 Conclusion

There is no doubt that each patient with a previous caesarean section has the right to choose TOLAC or ERCS. However, at the same time, she has the right to be fully informed and made aware of the risks and benefits for each mode of delivery by a well-trained obstetrician. This is because that the proper counselling and education, including checklists and informed consent are main factors that affected the patients’ choice toward the mode of delivery. Also, proper pain management is one intervention that might encourage patients to opt TOLAC because fear of pain was the main reason that patients requested ERCS instead of TOLAC.

This study was conducted according to the Strengthening the reporting of cohort studies in surgery (STROCSS) 2019 Guideline [40].

## Data availability

The datasets generated and analyzed during the current study are available from the corresponding author.

## Provenance and peer review

Not commissioned, externally peer reviewed.

## Ethics and patient consent

Written informed consent was obtained from the patients for publication. Institutional approval was obtained from the Institutional Review Board at Jordan University of Science and Technology (19/117/2018). This study was conducted in accordance with the Declaration of Helsinki.

## Ethical approval

Institutional approval was obtained from the Institutional Review Board at Jordan University of Science and Technology 19/117/2018.

## Funding

This research did not receive any specific grant from funding agencies in the public, commercial, or not-for-profit sectors.

## Author contribution

All authors contributed significantly and in agreement with the content of the article. All authors were involved in project design, data collection, analysis, statistical analysis, data interpretation and writing the manuscript. All authors presented substantial contributions to the article and participated of correction and final approval of the version to be submitted.

## Registration of research studies

researchregistry5369

https://www.researchregistry.com/browse-the-registry#home/?view_2_search=researchregistry5369&view_2_page=1.

## Guarantor

Dr. Amer Sindiani.

## Declaration of competing interest

The Authors declare that there is no conflict of interest.
