# Supplementary material for: Factors that influenced pregnant women with one previous caesarean section regarding their mode of delivery
Source: Ann Med Surg (Lond). 2020 May 18;55:124–30. doi: 10.1016/j.amsu.2020.05.007 (PMC7251298; doi:10.1016/j.amsu.2020.05.007)
Supplement: Multimedia component 1 [file mmc1.pdf]

| The STROCSS 2019 Guideline | | |
|---|---|---|
| **Item no.** | **Item description** | **Page** |
| **TITLE** | | |
| 1 | Title:<br>- The word cohort or cross-sectional or case-controlled is included<br>- The area of focus is described (e.g. disease, exposure/intervention, outcome)<br>- Key elements of study design are stated (e.g. retrospective or prospective) | 1 |
| **ABSTRACT** | | |
| 2a | Introduction: the following points are briefly described<br>- Background<br>- Scientific Rationale for this study | 1 |
| 2b | Methods: the following areas are briefly described<br>- Study design (cohort, retro-/prospective, single/multi-centred)<br>- Patient populations and/or groups, including control group, if applicable<br>- Interventions (type, operators, recipients, timeframes)<br>- Outcome measures | 1 |
| 2c | Results: the following areas are briefly described<br>- Summary data (with statistical relevance) with qualitative descriptions, where appropriate | 1 |
| 2d | Conclusion: the following areas are briefly described<br>- Key conclusions<br>- Implications to practice<br>- Direction of and need for future research | 2 |
| **INTRODUCTION** | | |
| 3 | Introduction: the following areas are described in full<br>- Relevant background and scientific rationale<br>- Aims and objectives<br>- Research question and hypotheses, where appropriate | 3 |
| **METHODS** | | |
| 4a | Registration and ethics<br>- Research Registry number is stated, in accordance with the declaration of Helsinki*<br>- All studies (including retrospective) should be registered before submission<br><br>*"*Every research study involving human subjects must be registered in a publicly accessible database before recruitment of the first subject*" (this can be obtained from: ResearchRegistry.com or ClinicalTrials.gov or ISRCTN) | N/A |
| 4b | Ethical Approval: the following areas are described in full<br>- Necessity for ethical approval<br>- Ethical approval, with relevant judgement reference from ethics committees<br>- Where ethics was unnecessary, reasons are provided | 4 |
| 4c | Protocol: the following areas are described comprehensively<br>- Protocol (*a priori* or otherwise) details, with access directions<br>- If published, journal mentioned with the reference provided | 5 |

| | | |
|---|---|---|
| 4d | Patient Involvement in Research<br>- Describe how, if at all, patients were involved in study design e.g. were they involved on the study steering committee, did they provide input on outcome selection, etc. | 4+5 |
| 5a | Study Design: the following areas are described comprehensively<br>- ‘Cohort’ study is mentioned<br>- Design (e.g. retro-/prospective, single/multi-centred) | 5 |
| 5b | Setting: the following areas are described comprehensively<br>- Geographical location<br>- Nature of institution (e.g. academic/community, public/private)<br>- Dates (recruitment, exposure, follow-up, data collection) | 5 |
| 5c | Cohort Groups: the following areas are described in full<br>- Number of groups<br>- Division of intervention between groups | 5 |
| 5d | Subgroup Analysis: the following areas are described comprehensively<br>- Planned subgroup analyses<br>- Methods used to examine subgroups and their interactions | 6 |
| 6a | Participants: the following areas are described comprehensively<br>- Eligibility criteria<br>- Recruitment sources<br>- Length and methods of follow-up | 5 |
| 6b | Recruitment: the following areas are described comprehensively<br>- Methods of recruitment to each patient group<br>- Period of recruitment | 4 |
| 6c | Sample Size: the following areas are described comprehensively<br>- Margin of error calculation<br>- Analysis to determine study population<br>- Power calculations, where appropriate | 6 |
| **INTERVENTION AND CONSIDERATIONS** | | |
| 7a | Pre-intervention Considerations: the following areas are described comprehensively<br>- Patient optimisation (pre-surgical measures)<br>- Pre-intervention treatment (hypothermia/-volaemia/-tension; ICU care; bleeding problems; medications) | N/A |
| 7b | Intervention: the following areas are described comprehensively<br>- Type of intervention and reasoning (e.g. pharmacological, surgical, physiotherapy, psychological)<br>- Aim of intervention (preventative/therapeutic)<br>- Concurrent treatments (antibiotics, analgaesia, anti-emetics, NBM, VTE prophylaxis)<br>- Manufacturer and model details where applicable | N/A |
| 7c | Intra-Intervention Considerations: the following areas are described comprehensively<br>- Administration of intervention (location, surgical details, anaesthetic, positioning, equipment needed, preparation, devices, sutures, operative time)<br>- Pharmacological therapies include formulation, dosages, routes and durations<br>- Figures and other media are used to illustrate | 5 |

| | | |
|---|---|---|
| 7d | Operator Details: the following areas are described comprehensively<br>- Training needed<br>- Learning curve for technique<br>- Specialisation and relevant training | 5 |
| 7e | Quality Control: the following areas are described comprehensively<br>- Measures taken to reduce variation<br>- Measures taken to ensure quality and consistency in intervention delivery | 5 |
| 7f | Post-Intervention Considerations: the following areas are described comprehensively<br>- Post-operative instructions and care<br>- Follow-up measures<br>- Future surveillance requirements (e.g. imaging, blood tests) | N/A |
| 8 | Outcomes: the following areas are described comprehensively<br>- Primary outcomes, including validation, where applicable<br>- Definitions of outcomes<br>- Secondary outcomes, where appropriate<br>- Follow-up period for outcome assessment, divided by group | 5 |
| 9 | Statistics: the following areas are described comprehensively<br>- Statistical tests, packages/software used, and interpretation of significance<br>- Confounders and their control, if known<br>- Analysis approach (e.g. intention to treat/per protocol)<br>- Sub-group analysis, if any | 6 |
| **RESULTS** | | |
| 10a | Participants: the following areas are described comprehensively<br>- Flow of participants (recruitment, non-participation, cross-over and withdrawal, with reasons)<br>- Population demographics (prognostic features, relevant socioeconomic features, and significant numerical differences) | 6-7 |
| 10b | Participant Comparison: the following areas are described comprehensively<br>- Table comparing demographics included<br>- Differences, with statistical relevance<br>- Any group matching, with methods | 7-10 |
| 10c | Intervention: the following areas are described comprehensively<br>- Changes to interventions, with rationale and diagram, if appropriate<br>- Learning required for interventions<br>- Degree of novelty for intervention | 7-10 |
| 11a | Outcomes: the following areas are described comprehensively<br>- Clinician-assessed and patient-reported outcomes for each group<br>- Relevant photographs and imaging are desirable<br>- Confounders to outcomes and which are adjusted | 7-10 |
| 11b | Tolerance: the following areas are described comprehensively<br>- Assessment of tolerance<br>- Loss to follow up, with reasons (percentage and fraction)<br>- Cross-over with explanation | N/A |
| 11c | Complications: the following areas are described comprehensively<br>- Adverse events described<br>- Classified according to Clavien-Dindo classification* | N/A |

| | | |
|---|---|---|
| | - Mitigation for adverse events (blood loss, wound care, revision surgery should be specified)<br><br>*Dindo D, Demartines N, Clavien P-A. Classification of Surgical Complications. A New Proposal with Evaluation in a Cohort of 6336 Patients and Results of a Survey. Ann Surg. 2004; 240(2): 205-213 | |
| 12 | Key Results: the following areas are described comprehensively<br>- Key results, including relevant raw data<br>- Statistical analyses with significance | 7-10 |
| **DISCUSSION** | | |
| 13 | Discussion: the following areas are described comprehensively<br>- Conclusions and rationale<br>- Reference to relevant literature<br>- Implications to clinical practice<br>- Comparison to current gold standard of care<br>- Relevant hypothesis generation | 10-12 |
| 14 | Strengths and Limitations: the following areas are described comprehensively<br>- Strengths of the study<br>- Limitations and potential impact on results<br>- Assessment of bias and management | 10-12 |
| 15 | Implications and Relevance: the following areas are described comprehensively<br>- Relevance of findings and potential implications to clinical practice are detailed<br>- Future research that is needed is described, with study designs detailed | 10-12 |
| **CONCLUSION** | | |
| 16 | Conclusions:<br>- Key conclusions are summarised<br>- Key directions for future research are summarised | 12 |
| **DECLARATIONS** | | |
| 17a | Conflicts of interest<br>- Conflicts of interest, if any, are described | 13 |
| 17b | Funding<br>- Sources of funding (e.g. grant details), if any, are clearly stated | 13 |
